# Mechanisms of actin disassembly and turnover

**DOI:** 10.1083/jcb.202309021

**Published:** 2023-11-10

**Authors:** Bruce L. Goode, Julian Eskin, Shashank Shekhar

**Affiliations:** 1 Department of Biology, Rosenstiel Basic Medical Science Research Center, Brandeis University, Waltham, MA, USA; 2 Departments of Physics, Cell Biology and Biochemistry, Emory University, Atlanta, GA, USA

## Abstract

Cellular actin networks exhibit a wide range of sizes, shapes, and architectures tailored to their biological roles. Once assembled, these filamentous networks are either maintained in a state of polarized turnover or induced to undergo net disassembly. Further, the rates at which the networks are turned over and/or dismantled can vary greatly, from seconds to minutes to hours or even days. Here, we review the molecular machinery and mechanisms employed in cells to drive the disassembly and turnover of actin networks. In particular, we highlight recent discoveries showing that specific combinations of conserved actin disassembly-promoting proteins (cofilin, GMF, twinfilin, Srv2/CAP, coronin, AIP1, capping protein, and profilin) work in concert to debranch, sever, cap, and depolymerize actin filaments, and to recharge actin monomers for new rounds of assembly.

## Teaser

Goode and colleagues review our current understanding of the cellular machinery and mechanisms driving actin network remodeling, disassembly, and turnover.

## Introduction

**Figure 1.**
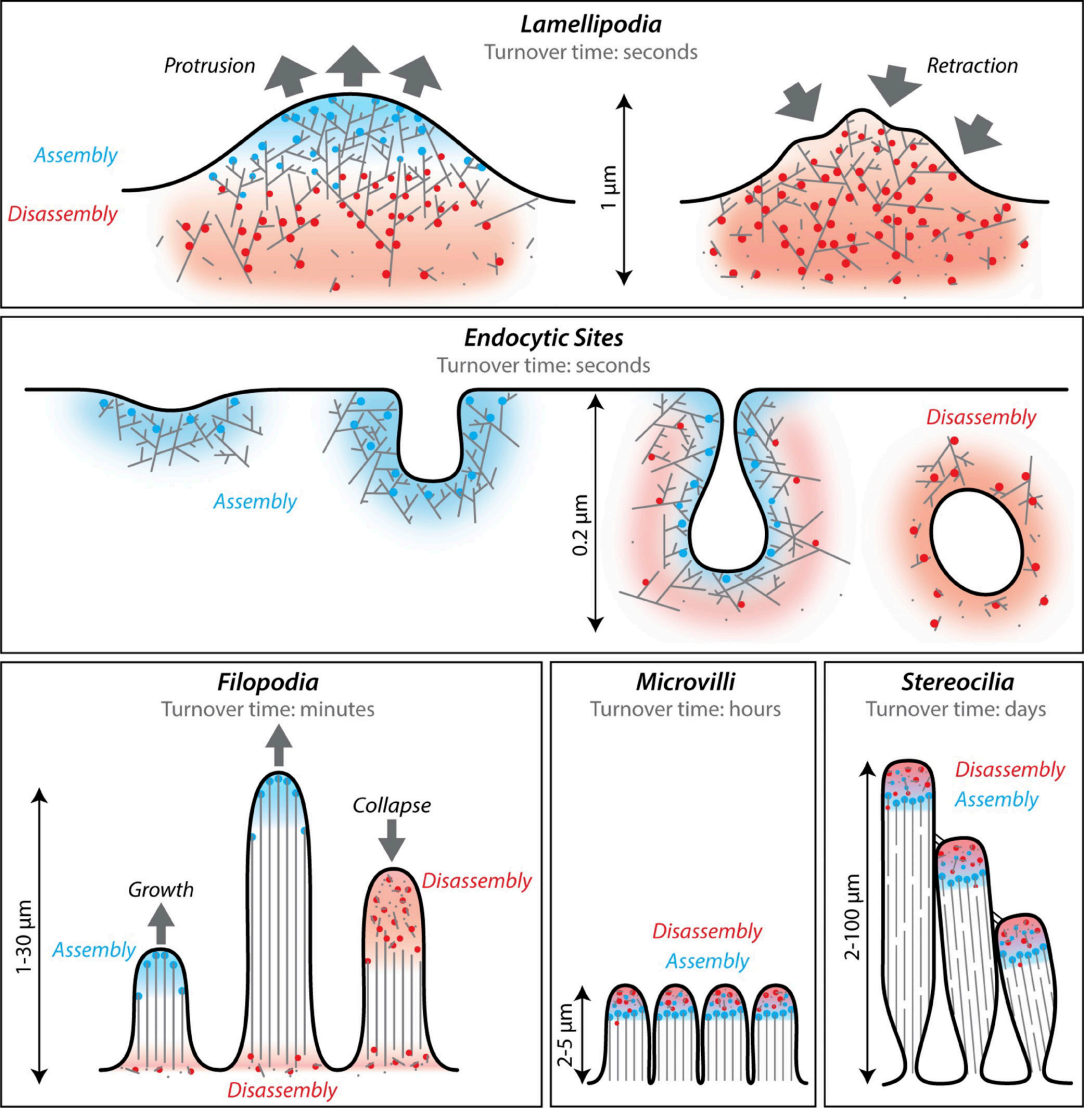
**Cellular actin structures with different filamentous architectures and turnover dynamics.** Panels show different types of in vivo actin networks and highlight the variation in their rates of turnover and filamentous architectures, as well as where new actin assembly occurs (blue) and where actin disassembly (red) occurs within each network. Blue dots, assembly-promoting factors. Red dots, disassembly-promoting factors. The upper row highlights lamellipodial protrusion and retraction. Protrusion (left) is driven by assembly of branched filaments, with disassembly occurring at the rear of the network. Retraction (right) is driven by attenuation of assembly coupled with network disassembly. At endocytic sites (middle row), branched filament assembly drives initial membrane invagination (left) and then a combination of assembly and disassembly drives vesiculation and scission (right). The lower row highlights formation and turnover of several different actin structures composed of unbranched filaments (filopodia, microvilli, and stereocilia). Filopodia are turned over by the demolition pathway, and thus grow until they abruptly collapse. In contrast, microvilli and stereocilia are regulated via the dynamic maintenance pathway, thus persisting as stable structures while their constituent actin filaments are continuously turned over.

Actin is one of the most abundant proteins in eukaryotic cells (>100 µM in vertebrate cells [Blikstad et al., 1978; Koestler et al., 2009]) and readily polymerizes into filaments. Actin filaments are spatially organized into elaborate networks with specific biological functions, ranging from muscle contraction and cell migration to endocytosis, intracellular transport, and cytokinesis. Depending on its cellular function, actin networks adopt highly variable shapes, sizes, and filamentous architectures. This includes branched actin arrays found at the cell’s leading edge and sites of endocytosis, tightly packed parallel bundles in filopodia and stereocilia, and antiparallel arrays in muscle sarcomeres, stress fibers, and cytokinetic actin rings (Fig. 1).

In the eukaryotic cytosol, a pool of actin monomers is maintained at high concentrations (10–200 µM), orders of magnitude above the critical concentration for filament assembly (0.12 µM; Wegner and Isenberg, 1983). This is achieved by a combination of cellular factors, including high concentrations of profilin and thymosin-β4 to bind monomers and capping protein (CP) to limit growth at filament barbed ends. By capping the majority of barbed ends in the cell, the pool of actin monomers builds up to high levels and is “funneled” to the small fraction of barbed ends that are uncapped, feeding their rapid growth (Loisel et al., 1999; Shekhar and Carlier, 2017).

Cytosolic conditions strongly favor actin assembly; therefore, specialized mechanisms are needed to destabilize filaments and drive their disassembly. Indeed, dynamic turnover of actin networks is crucial for their biological functions and gives cells the plasticity necessary to respond to signaling events and rapidly rearrange and/or dismantle actin networks. Turnover refers to the polarized flux of actin subunits through a filamentous actin network, where subunits are preferentially added at one end of the network and lost at the other. This polarity reflects the polarity of individual actin filaments, which are believed to assemble predominantly at the “barbed ends” and depolymerize at the “pointed ends” (Box 1). Filaments assembled from purified actin turn over slowly in vitro (one subunit every 3–4 s). However, pioneering work from Yu-li Wang showed that F-actin networks in living cells turn over orders of magnitude faster, suggesting that cells must have factors that catalyze actin disassembly (Box 2). In vivo, the turnover rates of different actin structures can vary greatly, from seconds for the networks at the leading edge and sites of endocytosis (Kaksonen et al., 2005; Lacy et al., 2019; Toshima et al., 2016; Watanabe and Mitchison, 2002) to hours or even days for stereocilia and sarcomeres (Narayanan et al., 2015; Rzadzinska et al., 2004; Wang et al., 2005; Fig. 1). How such a wide range of turnover rates is specified in vivo is only now beginning to be understood and appears to involve differential tuning of the actin disassembly mechanisms described below.

Box 1 Basics of actin filament nucleotide cycle and turnover

A unifying feature of actin networks is their polarity, which stems from the polarity of their constituent actin filaments. Actin filaments have a barbed end, where most of the growth occurs in vivo, and a pointed end, where most of the disassembly is thought to occur. The barbed and pointed ends of the filament have highly distinct kinetic properties, which now have been explained by important differences in the conformation of actin subunits at the two ends, as revealed by a high-resolution cryoEM structure of F-actin (Carman et al., 2023). The changing nucleotide state of actin subunits in the filament also plays an important role in its disassembly and turnover. When an ATP-actin monomer associates with the barbed end of the filament, its non-covalently bound ATP undergoes rapid hydrolysis (0.3 s^−1^), producing ADP-P_i_-actin subunits (Blanchoin and Pollard, 2002). In a much slower step (0.007 s^−1^), the inorganic phosphate (P_i_) dissociates, producing ADP-actin subunits (Carlier and Pantaloni, 1986; Carlier et al., 1987; Jégou et al., 2011). Actin filaments in these two different nucleotide states (ADP-P_i_ and ADP) have large differences in their kinetic properties, with ADP-actin subunits dissociating much faster than ADP-P_i_-actin subunits from filament ends (Galkin et al., 2010; Pollard, 1986). In this manner, a filament “ages” due to its nucleotide state. At steady state, filaments undergo treadmilling, where there is net growth at the barbed ends and net depolymerization at the pointed ends. The rate-limiting step in treadmilling is dissociation of ADP-actin subunits from the pointed ends (0.27 s^−1^; Pollard, 1986), and therefore, in the absence of additional cellular factors, filament turnover is relatively slow. However, actin structures in vivo often turnover at much faster rates (Fig. 1), as first revealed in the pioneering work of Yu-li Wang (Box 2). This now can be explained in large part by the activities of the actin disassembly-promoting factors highlighted in this review.

Box 2 Early discovery of rapid actin turnover in cells

Our understanding of cellular actin turnover took a major leap forward with the pioneering work of Yu-li Wang (Wang, 1984, 1985), who used live cell imaging to directly visualize actin turnover at the leading edge for the first time. Prior to this work, the turnover of F-actin in cells was presumed by many to be slow, based on the biochemical properties of filaments assembled from purified actin, which turn over (or treadmill) very slowly. The rate-limiting step in this turnover is the dissociation of subunits from the pointed ends of filaments (0.27 s^−1^). At this rate, a filament that is 1 μm long requires ∼15 min to turn over (Bonder et al., 1983; Pollard and Mooseker, 1981). However, Wang microinjected cells with fluorescent actin and monitored fluorescence recovery after photobleaching at the leading edge, observing a turnover rate of 0.8 μm/min, at least 10-fold faster than the turnover of purified filaments (Okabe and Hirokawa, 1989; Wang, 1985). While this study was met with initial skepticism raised by concerns that photodamage might be leading to the unexpectedly fast turnover, subsequent studies from Theriot and Mitchison observed similarly high rates of turnover by photoactivation of actin instead of bleaching, both at the leading edge and in *Listeria* tails (Theriot and Mitchison, 1991, 1992; Theriot et al., 1992). This was later confirmed at the leading edge by powerful fluorescence speckle microscopy observations, directly visualizing the turnover of individual GFP-actin molecules (Watanabe and Mitchison, 2002). As live-imaging techniques have steadily improved and become widely used, actin turnover has been examined for a broad range of cellular actin arrays in different organisms. Collectively, this work has demonstrated that the turnover rates of cellular actin structures vary tremendously, ranging from seconds or minutes to hours or days (see Fig. 1).

**Figure 2.**
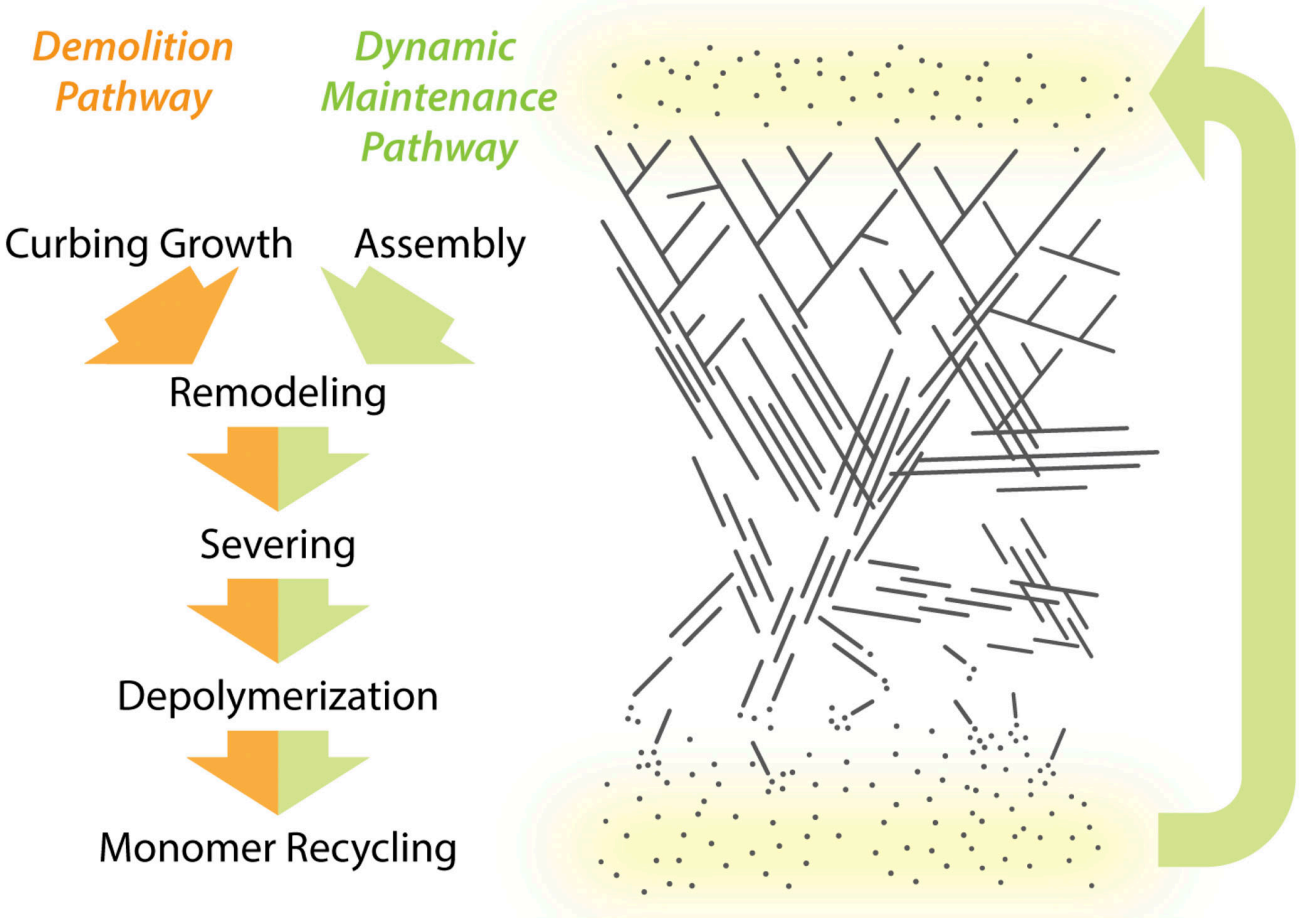
**Two pathways for cellular actin network turnover.** Actin networks in cells are either maintained in a state of polarized flux, where filaments undergo dynamic turnover (dynamic maintenance pathway), or they are targeted for net disassembly (demolition pathway). Both pathways involve actin filament remodeling, severing, depolymerization, and monomer recycling. In the dynamic maintenance pathway, assembly and disassembly are balanced. In the demolition pathway, network growth is severely curbed, which promotes network collapse due to filament disassembly mechanisms.

For the purpose of discussing the mechanisms that drive actin network turnover, we have deconstructed this process into five discrete steps. However, it is important to keep in mind that each step can occur simultaneously within a network. The steps are: (1) curbing barbed end growth, (2) pruning branched filaments, (3) severing filaments with concomitant capping of new barbed ends, (4) promoting depolymerization from filament ends, and (5) recycling ADP-actin monomers back to ATP-actin monomers for new rounds of assembly. Each step is driven by one or more molecular mechanisms and is used to either (a) maintain a network’s size and shape while turning over its constituent filaments, which we refer to as the “dynamic maintenance” pathway, or (b) induce net disassembly of the network, which we refer to as the “demolition” pathway (Fig. 2). Networks that undergo dynamic maintenance include microvilli, stereocilia, sarcomeres, and stress fibers, where the size and shape of the actin structure remain fairly constant, while its individual filaments continue to undergo turnover (Fig. 1). This phenomenon requires balanced assembly and disassembly within the network, regardless of whether the turnover rate is slow or fast. Networks that undergo demolition include a retracting filopodium, a retracting lamellipodia, and a cortical endocytic patch disassembling after its relatively short lifetime (Breitsprecher et al., 2011; Mallavarapu and Mitchison, 1999; Smith et al., 2001; Theriot and Mitchison, 1991; Wang, 1985; Fig. 1).

**Figure 3.**
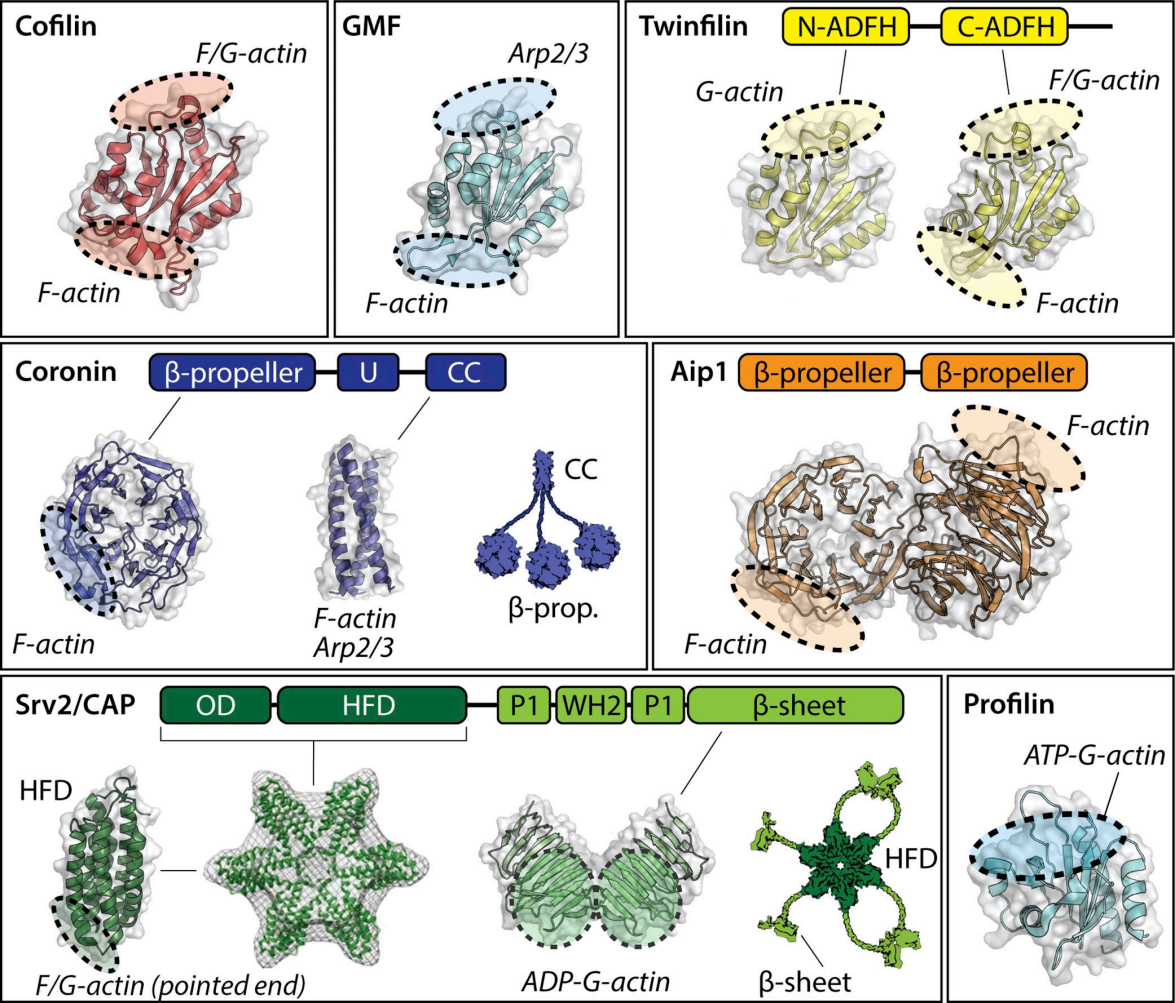
**Structures of proteins that promote actin turnover.** Each protein (or its domains) is shown as surface-rendered views with embedded cartoons of the secondary structural elements. Shaded areas indicate approximate positions of the binding sites for G-actin, F-actin, and Arp2/3 complex (color-coded). Cofilin and GMF share the ADF-H domain fold. Twinfilin consists of two ADF-H domains separated by a short linker and flanked by a C-terminal tail that binds CP. Coronins oligomerize via their coiled-coil (CC) domains, and use their β-propeller and CC domains to bind F-actin, and their unique (U) and CC domains to interact with Arp2/3 complex. EM reconstructions have shown that the N-terminal half of Srv2/CAP, consisting of the oligomerization domain (OD) and HFD, assembles into hexameric shurikens that bind to the sides and pointed ends of actin filaments. The C-terminal half of Srv2/CAP further consists of an actin-binding WH2 domain flanked by two proline-rich domains (P1 and P2) and an actin-binding β-sheet/CARP domain. P1 mediates interactions with profilin, while P2 binds to SH3 domain–containing proteins. PDB ID of structures: 4BEX (human cofilin-1), 1VKK (mouse GMF-γ), 1M4J (N-ADFH mouse TWF1), 3DAW (C-ADFH mouse TWF1), 1PGU (yeast AIP1), 2AQ5 (β-propeller domain of mouse coronin-1A), 2AKF (coiled-coil domain of mouse coronin-1A), 1S0P (HFD of *Dictyostelium* Srv2/CAP), 1K4Z (β-sheet/CARP domain of yeast Srv2/CAP), and 2PAV (human profilin-1, extracted). N-ADFH, N-terminal actin depolymerizing factor homology; C-ADHF, C-terminal actin depolymerizing factor homology.

One of the main purposes of this review is to summarize our current understanding of the conserved mechanisms regulating actin turnover and to highlight their molecular complexity. For many years, studies on cellular actin disassembly and turnover centered on the actions of only three proteins, actin depolymerizing factor (ADF)/cofilin, CP, and profilin (Loisel et al., 1999; Pollard and Borisy, 2003). CP would terminate barbed end growth, ADF/cofilin would sever and depolymerize filaments, and profilin would recycle actin monomers for new rounds of assembly. However, it has become clear that these proteins are not sufficient to drive rapid actin turnover in vivo. Instead, at least five other ubiquitous factors are required: glia maturation factor (GMF), twinfilin, Srv2/cyclase-associated protein (CAP), coronin, and AIP1. These eight proteins constitute a core actin disassembly-promoting machinery in cells, while additional factors found in different organisms and cell types are undoubtedly involved. Importantly, the eight proteins we focus on here are conserved from fungi to animals and expressed in all known animal cell types (Bertling et al., 2004; Cai et al., 2005; Vartiainen et al., 2002, 2003). Further, each protein has distinct activities and functional role(s) in driving actin disassembly and/or recycling (Fig. 3). Cofilin, CP, AIP1, and Srv2/CAP are additionally found in plants (Chaudhry et al., 2007; Inada, 2017; Shi et al., 2013). Further, some of these proteins are found in protists (Hliscs et al., 2010; Kallio and Kursula, 2014; Mehta and Sibley, 2010). For example, malaria-causing plasmodium expresses cofilin, profilin, coronin, CP, and a truncated version of CAP (Sattler et al., 2011), and flagellated *Leishmania* additionally expresses twinfilin (Kotila et al., 2022; Vizcaíno-Castillo et al., 2020). Notably, three of the eight proteins (ADF/cofilin, GMF, and twinfilin) are structurally related to each other as members of the ADF-homology (ADF-H) domain family (Poukkula et al., 2011). Below, we discuss how specific combinations of these proteins collaborate through multicomponent mechanisms to drive distinct steps in actin network turnover.

## Step 1: Curbing barbed end growth

In both the demolition and dynamic maintenance pathways, an important control point in network turnover is the attenuation of actin filament growth. This is achieved through proteins that cap the barbed ends of filaments (Fig. 2). Proteins that can either transiently or more stably cap barbed ends and block polymerization include CP, Eps8, adducin, gelsolin, twinfilin, villin, and IQGAP1 (Helfer et al., 2006; Hertzog et al., 2010; Hoeprich et al., 2022; Kuhlman et al., 1996; Mwangangi et al., 2021; Nag et al., 2013). Among these proteins, the activity of CP is the best understood (Edwards et al., 2014). CP is ubiquitously expressed, abundant in the cytosol (1–2 µM in yeast and mammalian cells; Fujiwara et al., 2014; Gonzalez Rodriguez et al., 2023; Kim et al., 2004), and arrests filament growth upon binding to the barbed end. In the absence of other cellular factors, CP remains stably bound to the barbed end, on average for tens of minutes. Through this activity, CP plays an important role in promoting the formation of actin networks comprised of short, branched filaments such as those found at the leading edge of motile cells and sites of endocytosis (Collins et al., 2011; Mullins et al., 1998; Rodal et al., 2005; Svitkina and Borisy, 1999; Young et al., 2004). Further, a recent study revealed that CP and the WH2 domains of Arp2/3 nucleation-promoting factors (NPFs) compete for filament barbed ends. This competition with CP liberates NPFs from barbed ends to bind actin monomers, indirectly stimulating Arp2/3-dependent branched actin nucleation (Funk et al., 2021). This mechanism may explain earlier observations showing that while CP limits the growth of individual filaments in networks, at the same time it promotes branched nucleation (Akin and Mullins, 2008).

**Figure 4.**
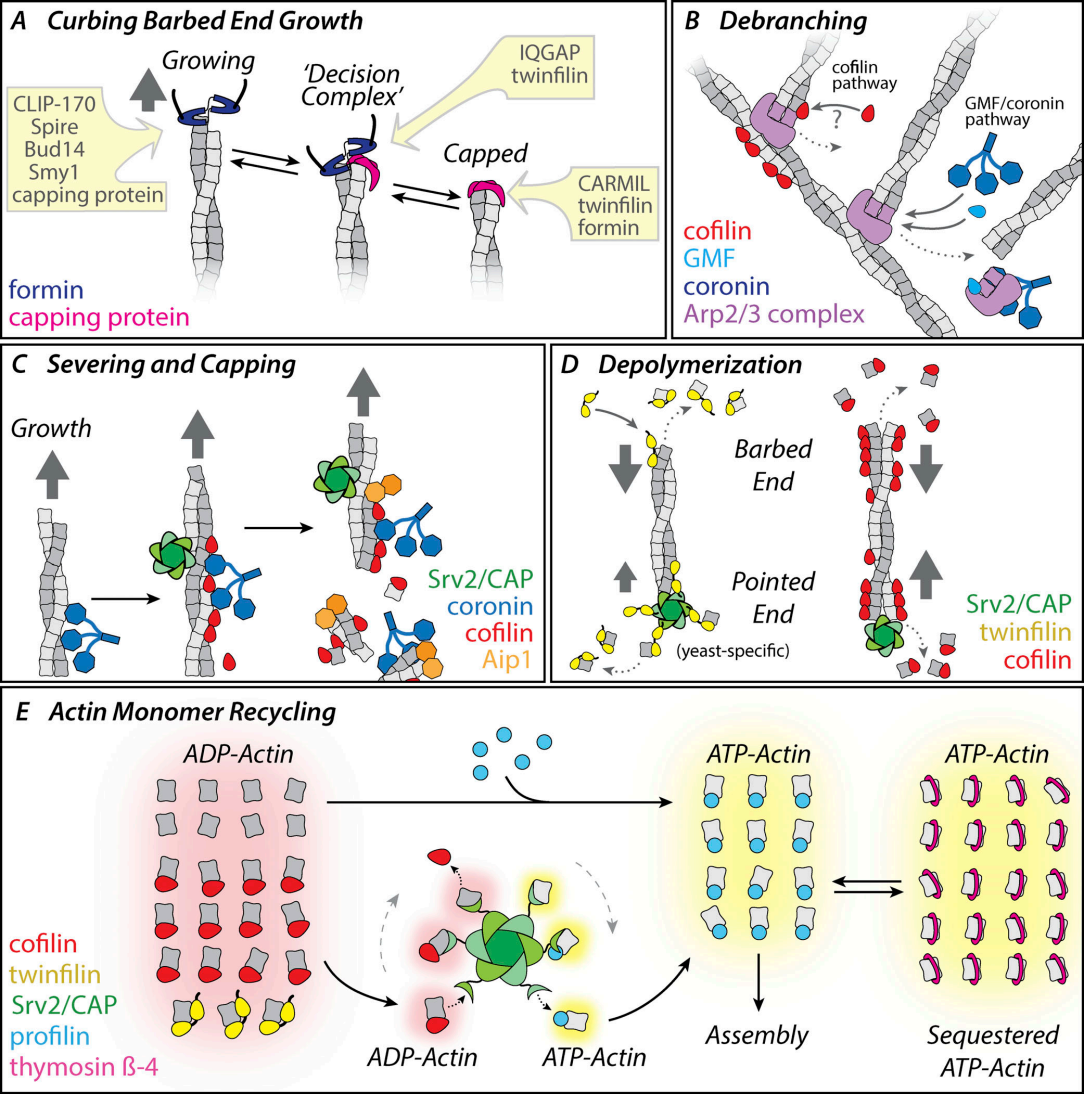
**Molecular mechanisms driving F-actin and G-actin turnover.** Each panel highlights a distinct step in actin network turnover. Proteins are color-coded. **(A)** Formins and CP join each other at the barbed ends of filaments to form decision complexes, and catalyze each other’s displacement. These transitions can be further accelerated by specific ligands of CP (e.g., twinfilin) and formins (e.g., IQGAP1), leading to rapid changes between states of filament growth and capping. Other ligands of CP and formins may influence their lifetimes at barbed ends. **(B)** Filament debranching mechanisms. The branch junctions nucleated by Arp2/3 complex are inherently stable, yet turn over rapidly in vivo. This is achieved by: (1) a mechanism involving cofilin binding to F-actin and/or Arp2/3 complex, and (2) mechanisms involving GMF and coronin, and their interactions with Arp2/3 complex. Debranching releases Arp2/3 complex, which can be strongly inhibited from nucleating actin assembly by GMF and coronin. **(C)** Filament severing and capping mechanisms. In the CCA mechanism, coronin binds to filaments first and recruits cofilin to these sites, increasing the efficiency of cofilin binding. Cofilin then recruits AIP1, which induces rapid severing. Severing produces new barbed ends, which are blocked from growth by CCA proteins. Filament severing can also be enhanced (4–10-fold) by a complementary mechanism in which Srv2/CAP and cofilin each bind independently to filament sides, and together accelerate severing. **(D)** Filament depolymerization mechanisms. At barbed ends, depolymerization can be accelerated by interactions with twinfilin or cofilin. At pointed ends, depolymerization can be accelerated by cofilin decoration of filament sides combined with processive association of Srv2/CAP with the pointed ends of filaments. Pointed end depolymerization also can be accelerated by Srv2/CAP and twinfilin, although the magnitude of the effects is species specific. **(E)** Regulation of the actin monomer pool. Filament disassembly releases ADP-actin monomers, which must be recycled for new rounds of assembly. Free ADP-actin monomers can bind profilin and rapidly exchange nucleotide (ATP for ADP). However, a large fraction of released ADP-actin monomers are bound to cofilin or twinfilin, which block nucleotide exchange and profilin binding. Srv2/CAP catalyzes the displacement of cofilin and twinfilin from the ADP-actin monomers, accelerates nucleotide exchange on G-actin, and hands off ATP-actin monomers to profilin. These activities stem from Srv2/CAP’s 100-fold higher affinity for ADP-actin (K_d_ = 18 nM) compared with ATP-actin (K_d_ = 1.8 µM). In vertebrate cells, high concentrations of ATP-actin monomers are maintained in a dynamic equilibrium between transient binding to thymosin-β4 (which keeps monomers in a sequestered state) and transient binding to profilin (which makes monomers available for assembly). Consumption of ATP-actin monomers by rapid filament assembly releases free profilin, which then rapidly replenishes the assembly-competent pool of profilin-bound ATP-actin monomers via dynamic competition with thymosin-β4.

In addition to its roles in governing the formation of branched actin networks, CP has been shown to join formins at the barbed ends of actin filaments in vitro to form transient “decision complexes” (Fig. 4 A; Bombardier et al., 2015; Shekhar et al., 2015). The formation of these complexes increases the rate of dissociation of both CP and formin from the barbed end by 10- to 50-fold. Moreover, the frequency of these transitions at the barbed end can be increased by ligands of CP (e.g., twinfilin) and formin (e.g., IQGAP1; Fig. 4 A; Pimm et al., 2023, *Preprint*; Ulrichs et al., 2023). Collectively, these associative competition mechanisms promote rapid transitions between states of barbed end growth and capping in vitro. In vivo, this competitive interplay between CP and formins is important for maintaining the characteristic size, architecture, and dynamics of cellular actin networks. For instance, in yeast cells the deletion of CP leads to enlargement of the cortical actin patches nucleated by Arp2/3 complex, which results in aberrant recruitment of formins and tropomyosins to cortical patches, altering patch dynamics (Billault-Chaumartin and Martin, 2019; Wirshing et al., 2023). Such interplay between CP and formins could also play an important role in filopodial extension. Filopodia grow by subunit addition at their distal tips, where they harbor formins and CP, which have been shown to have antagonistic roles in filopodia formation and dynamics (Mejillano et al., 2004; Mellor, 2010; Sinnar et al., 2014). After filopodia grow to reach suitable lengths, growth pauses and filopodia abruptly switch to a state of rapid disassembly and shortening (Fig. 1; Breitsprecher et al., 2011; Mallavarapu and Mitchison, 1999; Svitkina et al., 2003). These acute transitions from growth to demolition may involve displacement of formins from the barbed ends by CP, IQGAP1, and/or other proteins.

Additional layers of barbed end regulation are achieved by other ligands of CP. This includes the CP inhibitor capping protein, Arp2/3, and myosin I linker (CARMIL), which localizes to the leading edge and strongly catalyzes CP displacement from barbed ends in vitro. Through this activity, CARMIL regulates lamellipodial protrusion and cell polarity (Edwards et al., 2013; Fujiwara et al., 2014; Liang et al., 2009; Yang et al., 2005). Another abundant inhibitor of CP is V-1/myotrophin, which does not displace CP from barbed ends, but instead sequesters CP in an inactive state in the cytoplasm, helping to restrict CP activity to the leading edge (Bhattacharya et al., 2006; Hernandez-Valladares et al., 2010; Taoka et al., 2003; Yang et al., 2005).

A third CP ligand, twinfilin (Fig. 3), has multiple effects on the interactions of CP with the barbed end (Falck et al., 2004; Palmgren et al., 2001). The C-terminal tail of twinfilin contains a CP-interacting (CPI) motif through which it binds directly to CP with high affinity and competes with other CPI proteins like CARMIL (Johnston et al., 2018). Although CARMIL and twinfilin both displace CP from barbed ends, the uncapping activity of CARMIL is much stronger than that of twinfilin (180-fold versus sixfold faster dissociation of CP; Fujiwara et al., 2010; Hakala et al., 2021). This difference, together with the observation that CARMIL is restricted to the plasma membrane while twinfilin is found throughout leading edge actin networks and in the cytosol, suggests that they may have spatially distinct roles in regulating CP. Additional insights into the twinfilin-CP mechanism have come from a crystal structure of twinfilin bound to CP and two actin subunits (Mwangangi et al., 2021), and from a recent single molecule study showing that twinfilin molecules join CP at the barbed end in many repeated, short-lived interactions before CP displacement occurs (Ulrichs et al., 2023).

In addition to its roles in leading edge actin networks, twinfilin localizes to endocytic actin patches and the tips of filopodia and stereocilia (Goode et al., 1998; Palmgren et al., 2001; Rzadzinska et al., 2004; Ydenberg et al., 2015). Genetic observations in some of these studies have shown that twinfilin regulates the size and turnover of these actin-based structures, which may result from twinfilin functions at the barbed ends of filaments. Importantly, twinfilin not only promotes CP dissociation but also has important roles in driving depolymerization at filament ends, as described below (Fig. 4, A and D).

Together, these observations suggest that a large number of cellular factors dynamically compete for filament ends and/or interact with each other at filament ends, collectively attenuating barbed end growth.

## Step 2: Pruning branched filaments

Following their assembly, the shape and filamentous architecture of actin networks can be rapidly transformed or “remodeled.” Such transitions are brought about through filament debranching, severing, and crosslinking mechanisms. In this section, we focus on debranching, which is specific to actin networks assembled by the branch-nucleating Arp2/3 complex. Initially, these actin structures are assembled as densely branched arrays, optimized for exerting force on membranes to drive protrusion and/or invagination (Rotty et al., 2013). However, as the filaments within the networks age, they move inward from the cortex due to continued polymerization at the cortex and actomyosin retrograde flow (Watanabe and Mitchison, 2002). As the networks move in, filaments are pruned, i.e., daughter branches dissociate from mother filaments (see Fig. 3 in Ydenberg et al., 2011; Urban et al., 2010; Yang and Svitkina, 2011; Ydenberg et al., 2011). In vivo, it is likely that debranching contributes not only to network remodeling but also to network disassembly, as it exposes (or uncaps) pointed ends of daughter filaments. In vitro, the filament branch junctions formed by Arp2/3 complex are extremely stable, persisting for tens of minutes, whereas in vivo they can turn over in 2–30 s (Lacy et al., 2019; Miyoshi et al., 2006). This disparity in rates in vitro versus in vivo suggests that cells have mechanisms for catalyzing debranching. To date, three proteins have been strongly implicated in promoting debranching: cofilin, coronin, and GMF.

Cofilin catalyzes debranching in addition to its more well-established roles in actin filament severing and depolymerization (Blanchoin et al., 2000; Chan et al., 2009; Chung et al., 2022; Fig. 4 B). Importantly, cofilin shows distinct concentration dependencies in its debranching and severing activities, suggesting that debranching occurs by a distinct mechanism. Recent total internal reflection fluorescence (TIRF) studies using labeled Arp2/3 complex verify that cofilin induces debranching (which removes Arp2/3 complex) rather than severing near branch junctions that would leave Arp2/3 complex on the filament side (Chung et al., 2022). The same study also showed that debranching events are preceded by the appearance of labeled cofilin somewhere close to the branch junction. Despite these advances, the molecular mechanism by which cofilin induces debranching remains poorly understood and merits further investigation.

A second protein implicated in debranching is coronin. Mammals have seven coronin genes, whereas yeast has only one (Fig. 4 B). Yeast coronin and several of the mammalian coronin isoforms bind Arp2/3 complex in addition to F-actin (Fig. 3; Cai et al., 2008; Föger et al., 2006; Gandhi et al., 2009; Goode et al., 1999; Humphries et al., 2002; Spoerl et al., 2002). Most coronin isoforms oligomerize and crosslink actin filaments in vitro (Gatfield et al., 2005; Goode et al., 1999; Oku et al., 2005; Spoerl et al., 2002). A role for coronin in debranching was first reported when depletion of mammalian coronin-1B was found to increase the density of branched filaments and barbed ends close to the leading edge and alter lamellipodial dynamics (Cai et al., 2007, 2008). Further, purified coronin-1B increased the in vitro debranching rate, although the underlying mechanism is still unclear. More recently, it was shown that coronin-7, which has a domain structure distinct from other coronin isoforms, also promotes debranching (Xie et al., 2021). It will be important to define the mechanism underlying these effects and determine whether debranching activities extend to coronin homologs in other model organisms.

The third protein implicated in debranching is GMF (Goode et al., 2018). GMF was first isolated from brain extracts and reported to be a cellular differentiation factor (Lim et al., 1977), but then was discovered to be a member of the ADF-H protein family and a component of the actin cytoskeleton involved in cell motility and endocytosis (Fig. 3; Gandhi et al., 2010; Goroncy et al., 2009; Ikeda et al., 2006). Biochemical analysis of yeast GMF revealed that it does not bind to actin, but instead has high affinity (K_d_ = 10 nM) for Arp2/3 complex (Gandhi et al., 2010; Ydenberg et al., 2013; Fig. 4 B). Through this direct interaction, yeast and mammalian GMF isoforms induce debranching in vitro and suppress Wiskott Aldrich syndrome protein (WASP) and Arp2/3 complex–mediated branched actin nucleation (Gandhi et al., 2010; Ydenberg et al., 2013). Consistent with these in vitro activities on Arp2/3 complex, overexpression of GMF in yeast leads to a reduction in the number of cortical actin patches and a concomitant increase in actin cables (Nakano et al., 2010). Knockdown of *Drosophila* GMF disrupts lamellipodial retraction and causes defects in border cell migration (Poukkula et al., 2014). Further, Bear and colleagues utilized a point mutation in GMF (*gmf1-101*), which disrupts its debranching activity but not its ability to inhibit branched nucleation (Ydenberg et al., 2013), to demonstrate that GMF’s debranching activity is required for normal Arp2/3 turnover rate at the leading edge and for proper lamellipodial protrusion and retraction dynamics (Haynes et al., 2015).

How then does GMF destabilize the branch junction? GMF makes two important molecular contacts that induce debranching, one with Arp2/3 complex and one with the first actin subunit in the daughter filament (Luan and Nolen, 2013; Ydenberg et al., 2013). The Arp2/3-binding site on GMF occupies the same position in the ADF-H fold as the F/G-actin–binding site on cofilin, whereas the daughter filament-binding site on GMF spatially corresponds to the F-actin–binding site on cofilin (Fig. 3; Goroncy et al., 2009; Luan and Nolen, 2013). Thus, the mechanisms by which GMF induces debranching and cofilin induces severing appear to be structurally related. In contrast, the mechanism by which cofilin induces debranching is not well understood.

Another important step in debranching is ATP hydrolysis and phosphate release from the Arp2 and Arp3 subunits of the Arp2/3 complex. These changes in nucleotide state are set in motion by nucleation of the daughter filament and are required for proper actin network turnover in vivo (Ingerman et al., 2013; Martin et al., 2005, 2006). Whether coronin, cofilin, and/or GMF promote debranching in part by altering the rates of ATP hydrolysis and/or phosphate release on Arp2/3 complex needs to be investigated. Interestingly, GMF exhibits higher affinity for ADP- compared to ATP-bound Arp2/3 complex (Boczkowska et al., 2013) and more effectively destabilizes aged branches (Pandit et al., 2020), so there is potential for multiple debranching factors working in concert to catalyze debranching. Finally, mechanical force can promote daughter filament detachment and possibly contribute to each of the biochemical debranching mechanisms above (Pandit et al., 2020).

One of the next challenges is to determine how the activities of these three debranching factors (cofilin, coronin, and GMF) are coordinated in vivo and how their activities are counterbalanced by known branch stabilizers such as Abp1 and cortactin. Yeast coronin and GMF have distinct binding sites on the Arp2/3 complex and synergistically inhibit Arp2/3-mediated actin nucleation (Sokolova et al., 2017). Further, yeast GMF and cofilin have additive debranching effects, suggesting that they destabilize branches via distinct and complementary mechanisms (Chung et al., 2022). Yeast Abp1, which is another conserved ADF-H family protein, competes with GMF for Arp2/3 complex binding and antagonizes the debranching activity of GMF (Guo et al., 2018). While cortactin has long been known to stabilize branches against spontaneous debranching (Helgeson and Nolen, 2013; Uruno et al., 2001; Weaver et al., 2002), it is unknown how it affects branch stability in the presence of branch destabilizers. Thus, there is much to learn about how branch stability is tuned in vivo through competitive interplay between branch destabilizers and stabilizers.

## Step 3: Filament severing and capping

A central mechanism cells use to promote actin network disassembly is severing, which amplifies the number of filament ends (barbed and pointed) from which subunits can dissociate. However, because the cytosol contains high concentration of actin monomers, severing without concomitant barbed end capping can instead stimulate F-actin assembly (Bravo-Cordero et al., 2013; Ghosh et al., 2004). Indeed, severing by cofilin amplifies the number of barbed ends and in the presence of actin monomers promotes F-actin assembly in vitro and at the leading edge of cells (Chan et al., 2000; Jansen et al., 2015; Mabuchi, 1983). However, when severing is accompanied by rapid capping of the newly generated barbed ends it strongly increases the rate of F-actin disassembly. Barbed end cappers that may be involved in this process in vivo include CP, gelsolin, Eps8, twinfilin, WAVE1, adducins, villin, and IQGAP1 (Edwards et al., 2014; Hoeprich et al., 2022; Pelikan-Conchaudron et al., 2011; Shekhar et al., 2016).

Across the animal, plant, and fungal kingdoms, cofilin is the most abundant and well-characterized severing protein, although other proteins with severing activity may also be involved, e.g., gelsolin, villin, spire, cordon-bleu, and specific formins (Bosch et al., 2007; Chhabra and Higgs, 2006; Harris et al., 2004; Heimsath and Higgs, 2012; Husson et al., 2011; Nag et al., 2013). Mammals have three distinct cofilin genes which encode cofilin-1, ADF, and cofilin-2. Cofilin-1 is ubiquitously expressed, ADF is found primarily in neuronal, epithelial, and endothelial tissues, and cofilin-2 is expressed in heart and skeletal muscle tissue (Vartiainen et al., 2002). All three cofilins sever filaments and promote actin disassembly to different extents in vitro (Chin et al., 2016; Kremneva et al., 2014). In addition, all three cofilins promote depolymerization at filament ends in vitro (see next section). For the remainder of the review, we refer to cofilin proteins collectively as “cofilin” in order to focus on their shared properties; however, in a few instances we refer to their specific names to highlight differences.

Cofilins are essential for a wide range of actin-based processes that require rapid actin turnover, e.g., endocytosis, cell migration, cytokinesis, and cell morphogenesis (Chen et al., 2001; Ghosh et al., 2004; Lappalainen and Drubin, 1997; Lin et al., 2010; Loisel et al., 1999; Nakano and Mabuchi, 2006; Okreglak and Drubin, 2007). The mechanism by which cofilin severs filaments is well studied and has been reviewed elsewhere (Elam et al., 2013a; Hild et al., 2010; Poukkula et al., 2011). In brief, cofilin binds preferentially to “aged” ADP-actin (monomeric and filamentous), but also catalyzes P_i_ release from ADP-P_i_ subunits in filaments, possibly via allosteric effects (Blanchoin and Pollard, 1999). Cofilin uses two distinct actin-binding surfaces (Fig. 3) to bind filaments cooperatively and dramatically alters F-actin conformation and mechanical properties. This leads to local discontinuities between the cofilin-decorated and “bare” regions on a filament, promoting breaks (Elam et al., 2013b; Galkin et al., 2011; Kang et al., 2014; Lappalainen et al., 1997; Suarez et al., 2011). TIRF microscopy and high-speed atomic force microscopy (AFM) studies further show that multiple cofilin molecules form a cluster on filament sides, which leads to conformational effects that propagate toward the pointed end (Gressin et al., 2015; Ngo et al., 2015). However, high-resolution electron microscopy indicates that the most obvious conformational changes in F-actin are restricted to the subunits where cofilin is bound (Huehn et al., 2018). Thus, further structural work is needed to resolve whether cofilin perhaps has more subtle and longer-range allosteric effects on F-actin that influence filament dynamics and/or interactions of other actin-binding proteins. Cofilin severing also can be enhanced by torsional stresses imposed by filament cross-linkers and/or contractile forces induced by myosin motors (Bibeau et al., 2023; Wioland et al., 2019b).

On its own, cofilin has several limitations as a disassembly-promoting factor, and its activities change considerably in the presence of its cellular co-factors AIP1, Srv2/CAP, and coronin. The first limitation is that cofilin binds to F-actin slowly, with an on-rate of only ∼10^5^ s^−1^ M^−1^ (Andrianantoandro and Pollard, 2006). Second, filaments extensively decorated by cofilin alone do not sever. On its own, cofilin only severs filaments efficiently at nanomolar concentrations, where decoration is sparse (Andrianantoandro and Pollard, 2006; Suarez et al., 2011). Third, as mentioned earlier, severing amplifies the number of barbed ends, and can therefore promote F-actin assembly rather than disassembly if the newly generated barbed ends are not rapidly capped. Importantly, each of these limitations is overcome by cofilin working with AIP1, coronin, and Srv2/CAP (Fig. 4 C). The structures of these three proteins and their actin-binding surfaces are highly conserved (Fig. 3), and their activities and mechanisms are summarized below. More information can also be found in reviews that focus on each protein (Chan et al., 2011; Gandhi and Goode, 2008; Ono, 2013, 2018).

Among the three co-factors, AIP1 is the only one known to directly bind cofilin. In vitro studies employing bulk and TIRF assays have shown that AIP1 works with cofilin to accelerate F-actin severing, and possibly pointed end depolymerization, both at the scale of single filaments and reconstituted actin networks (Clark et al., 2006; Gressin et al., 2015; Jansen et al., 2015; Nadkarni and Brieher, 2014; Okada et al., 1999, 2006; Rodal et al., 1999). Further, filaments heavily decorated by cofilin are rapidly severed in the presence of AIP1. Thus, AIP1 and cofilin together overcome one of the above-mentioned limitations of cofilin alone. These in vitro effects are strongly supported by genetic interactions between cofilin and AIP1 and by the in vivo observation that loss of *AIP1* leads to cofilin-decorated hyper-stabilized actin cables in yeast (Ishikawa-Ankerhold et al., 2010; Okada et al., 2006; Rodal et al., 1999; Ydenberg et al., 2015). Importantly, electron microscopy has revealed that in vitro AIP1 and cofilin together greatly reduce the size of F-actin severing products compared to cofilin alone (Jansen et al., 2015; Okada et al., 1999). Whereas cofilin alone severs filaments into fragments ∼300–500 nm in length, AIP1 and cofilin together produce fragments only ∼50 nm long. Thus, AIP1 not only enhances the kinetics of severing, but also the extent of fragmentation.

Even more impressive than cofilin-AIP1 synergy is the combined activity of the coronin, cofilin, and AIP1 (or “CCA”) trio. Pioneering work from Brieher and colleagues identified these three proteins in crude mammalian cell extracts as factors that work together to drive rapid disassembly of *Listeria* actin tails even under assembly-promoting conditions (Brieher et al., 2006). Subsequent single molecule analysis showed that the CCA mechanism proceeds by an ordered pathway (Jansen et al., 2015; Fig. 4 C). In the first step, coronin binds to F-actin. Next, coronin recruits cofilin to the same sites on the filament, accelerating cofilin accumulation on filament sides. Finally, AIP1 joins cofilin at these sites, and immediately induces severing. In this manner, the CCA mechanism severs filaments with much higher efficiency than cofilin alone. Moreover, all three proteins remain at or near the newly generated barbed ends and block new growth. High concentrations of AIP1 and cofilin together are also sufficient to suppress barbed end growth in vitro even in the absence of coronin (Balcer et al., 2003; Okada et al., 2002; Rodal et al., 1999), and this function is supported by genetic interactions between AIP1 and CP (Balcer et al., 2003; Berro and Pollard, 2014). However, further addition of coronin enhances these activities of cofilin and AIP1, leading to tight suppression of growth at newly generated barbed ends (Jansen et al., 2015). CCA also has been reported to induce “catastrophic bursting” (the rapid disappearance of long stretches of F-actin from filament ends; Kueh et al., 2008; Tang et al., 2020). This potentially exciting mechanism merits further exploration, including determination of whether it involves mechanistic steps distinct from enhanced severing and depolymerization.

TIRF studies have shown that the three human cofilin isoforms have quantitative differences in their severing and depolymerization activities, yet all are enhanced by mammalian coronin and AIP1 (Chin et al., 2016). Further, yeast coronin (Crn1) accelerates yeast cofilin recruitment to filament sides, and yeast AIP1 strongly enhances cofilin-mediated severing and disassembly (Chen et al., 2015; Mikati et al., 2015; Okada et al., 2006; Rodal et al., 1999). Therefore, CCA activities appear to be conserved. However, it is still unclear whether mammalian coronin isoforms besides coronin-1B participate in multi-component disassembly mechanisms, and how similar or different they are from each other. Another unresolved issue is the structural basis for CCA synergy. While progress has been made in determining the electron microscopy structures of F-actin decorated individually by cofilin or coronin (Galkin et al., 2008, 2011; Ge et al., 2014), a deeper understanding will require high-resolution structures of filaments decorated by all three proteins. Dissection of such a complex mechanism may also benefit from the numerous separation-of-function mutants available in each of these proteins (Clark et al., 2006; Gandhi et al., 2010; Ge et al., 2014; Lappalainen et al., 1997; Mohri et al., 2004; Okada et al., 2006). While there are many details of the CCA mechanism yet to be resolved, it has served as an elegant illustration of how multiple cellular factors work together to produce biological effects that are not observed for any individual components.

A third co-factor that works with cofilin is CAP, which is called Srv2 in yeast (Balcer et al., 2003; Moriyama and Yahara, 2002; Ono, 2013). This 57-kD protein oligomerizes to form several distinct high-molecular-weight species seen by metal-shadowing electron microscopy (Balcer et al., 2003). Subsequent negative-stain single particle averaging studies revealed that the N-terminal halves of yeast and mouse CAP (N-CAP) form hexameric *shuriken* structures with six actin-binding “blades,” each comprised of a helical-folded domain (HFD; Fig. 3; Chaudhry et al., 2013; Jansen et al., 2014; Ksiazek et al., 2003; Yusof et al., 2005). However, more recent analyses of *Xenopus* and mouse CAP proteins, using analytical ultracentrifugation and AFM, suggest that CAP may form tetramers (Kodera et al., 2021; Purde et al., 2019). Thus, CAP may be capable of adopting distinct oligomeric states, possibly with different activity states.

CAP has multiple domains and in vitro activities in promoting actin turnover (Fig. 4, C–E). The C-terminal half of CAP (C-CAP) dimerizes and recycles actin monomers, stimulating nucleotide exchange (ATP for ADP) for new rounds of actin assembly. N-CAP oligomerizes and binds autonomously to the sides of actin filaments to enhance cofilin-mediated severing by four- to eightfold, but has no severing effects on its own (Chaudhry et al., 2013; Jansen et al., 2014; Normoyle and Brieher, 2012). The enhanced severing activity of N-CAP depends on a conserved F-actin–binding surface on its HFD (Fig. 3) and its oligomerization domain (Chaudhry et al., 2013; Jansen et al., 2014). Moreover, mutations at these sites cause in vivo defects in actin organization and endocytosis (Chaudhry et al., 2013; Jansen et al., 2014; Toshima et al., 2016). Importantly, CAP does not interact directly with cofilin, and unlike coronin it does not enhance cofilin recruitment to filament sides. Instead, CAP reduces the time between cofilin binding and severing (Chaudhry et al., 2013). Thus, CAP and coronin appear to have distinct, complementary roles in enhancing cofilin-mediated F-actin disassembly.

N-CAP also has strong effects in accelerating pointed end depolymerization, particularly in combination with cofilin (see next section for details). These effects also depend on the HFD (Kotila et al., 2019; Shekhar et al., 2019). Kotila and colleagues showed that the HFD binds to surfaces on actin exposed at the pointed ends of filaments, which led them to call into question whether N-CAP is capable of binding to filament sides or enhancing cofilin-mediated severing. However, multiple lines of evidence have demonstrated that N-CAP binds to filament sides: (1) In co-sedimentation assays, N-CAP pellets with F-actin (K_d_ ∼2 µM; Jansen et al., 2014). (2) In the same study, N-CAP was directly visualized on filament sides by EM, and induced “untwisting” of F-actin (Jansen et al., 2014). (3) Further, N-Srv2 and N-CAP enhanced cofilin-mediated severing in three separate open-flow TIRF studies from two different groups (Chaudhry et al., 2013; Jansen et al., 2014; Normoyle and Brieher, 2012). In contrast, Kotila and co-workers found that N-CAP failed to stimulate cofilin-mediated severing in microfluidic-TIRF experiments (Kotila et al., 2019). However, flow-induced tension on filaments may have altered N-CAP binding. Thus, further investigation is needed to fully resolve whether CAP associates with filament sides and enhances cofilin-mediated severing, and how these interactions might be influenced by filament tension.

Another cellular factor that works with cofilin to promote actin network disassembly is the enzyme Molecules Interacting with CasL (MICAL; for details, see Box 3). Briefly, when MICAL binds to F-actin, it is activated, resulting in oxidation of two conserved methionine residues in the D-loop of actin (subdomain II). TIRF microscopy studies show that oxidation of actin by MICAL promotes severing as well as depolymerization at both ends of the filament, and that these activities are synergistic with those of cofilin (Grintsevich et al., 2016, 2017, 2021; Hung et al., 2011; Wioland et al., 2021).

Box 3 Actin filament destabilization by PTM

An important but often overlooked mechanism in actin network turnover is destabilization of filaments by PTM of actin, induced by either cellular enzymes or bacterial toxins. The best-understood PTM that governs actin turnover is oxidation of Met44 in the D-loop of actin by the redox protein MICAL. Modification of even a small fraction of subunits in a filament leads to severing and/or depolymerization (Frémont et al., 2017; Hung et al., 2011). Further, these effects are synergistic with cofilin-mediated severing in promoting rapid filament turnover in vitro and in vivo (Grintsevich et al., 2016). The covalently modified actin monomers released by these mechanisms are compromised for assembly (Grintsevich et al., 2016), but can be restored to an assembly-competent state by SelR, a methionine sulfoxide reductase enzyme that reverses oxidation of Met44 on actin (Hung et al., 2013). Thus, through activation and inactivation of MICAL and SelR, cells can rapidly and reversibly remodel their actin cytoskeletons in response to cues. These activities of MICAL are important for cytokinesis, axon guidance, and normal cellular actin dynamics (Frémont et al., 2017; Giridharan et al., 2012; Grigoriev et al., 2011; Hung et al., 2010). In addition, several bacterial pathogens induce disassembly of their host cell’s actin cytoskeleton by producing toxins that modify actin subunits through ADP-ribosylation or covalent crosslinking (Aktories et al., 2011; Kudryashova et al., 2012). Further, there are normal mechanisms in eukaryotic cells for introducing similar modifications, e.g., transglutaminases, which crosslink actin subunits, and transferase A, which ADP-ribosylates actin (Del Duca et al., 2009; Dolge et al., 2012; Okamoto et al., 1997). Crosslinking of actin monomers leads to an accumulation of polymerization-deficient dimers, trimers, and oligomers, depleting the monomer pool and causing the disassembly of F-actin structures. Depending on the specific residue modified, ADP-ribosylation of actin differentially affects actin polymerization. For instance, ADP-ribosylation of Arg177 compromises polymerization and blocks barbed end growth, lowering F-actin levels, whereas ADP-ribosylation of Thr148 leads to higher F-actin levels, possibly by weakening thymosin-β4 interactions with the modified actin monomers (Hochmann et al., 2006; Perieteanu et al., 2010; Vandekerckhove et al., 1988; Wegner and Aktories, 1988; Weigt et al., 1989). Additional modifications of actin that can occur in vivo and can influence actin turnover include arginylation, acetylation, phosphorylation, methylation, and nitrosylation (Karakozova et al., 2006; Terman and Kashina, 2013). Collectively, these PTMs are opening new avenues for actin regulation research and ultimately must be considered alongside the multicomponent disassembly mechanisms described herein.

Collectively, the examples discussed above emphasize how cells employ multi-component molecular mechanisms to promote the rapid severing, capping, and depolymerization of actin filaments.

## Step 4: Accelerated depolymerization

In both the demolition and dynamic maintenance pathways, the depolymerization of filaments is a critical step in network turnover (Fig. 2). In the demolition pathway, the F-actin fragments produced by severing must be depolymerized into monomers, so that they can be recycled for use in new rounds of actin assembly. In the dynamic maintenance pathway, actin arrays of a specified length and architecture (e.g., microvilli or stereocilia) are maintained dynamically, due to balanced polymerization at one end of the network and depolymerization at the other end.

In the absence of cellular factors, the rate of subunit dissociation from pointed ends is slow (0.27 s^−1^; Pollard, 1986), and at this rate even the shortest fragments generated by cofilin and AIP1 severing (∼20 subunits long; Jansen et al., 2015; Okada et al., 1999) would still require over 1 min to depolymerize. Such slow rates are incompatible with the fast speeds of actin turnover observed in vivo for many actin networks (Fig. 1), suggesting that cells are likely to have mechanisms for accelerating subunit dissociation from filament ends. However, this concept was vigorously debated for many years, due primarily to a lack of direct observation of such effects. However, the debate was finally settled by a series of in vitro TIRF microscopy studies in 2015–2019, which directly visualized accelerated depolymerization at filament ends induced by twinfilin, cofilin, and/or Srv2/CAP (Fig. 4 D; Hilton et al., 2018; Johnston et al., 2015; Kotila et al., 2019; Shekhar and Carlier, 2017; Shekhar et al., 2019, 2021; Wioland et al., 2017).

The first of these studies showed that yeast Srv2/CAP and twinfilin jointly accelerate filament depolymerization at barbed ends (by approximately threefold) and pointed ends (by ∼20-fold; Johnston et al., 2015). Twinfilin is a conserved member of the ADF-H domain protein family, with an unusual molecular architecture consisting of two ADF-H domains separated by a short linker, followed by a C-terminal tail that interacts with capping protein (Fig. 3; Goode et al., 1998; Lappalainen et al., 1998; Palmgren et al., 2001). Twinfilin binds with high affinity to ADP-actin monomers and very low affinity to filament sides (Goode et al., 1998; Johnston et al., 2015; Ojala et al., 2002; Paavilainen et al., 2007; Vartiainen et al., 2000). Early genetic interactions between twinfilin and cofilin mutants had suggested a role for twinfilin in promoting actin turnover; however, for many years the only known activities of twinfilin were actin monomer sequestering and a poorly understood barbed end capping effect (Goode et al., 1998; Helfer et al., 2006; Vartiainen et al., 2000). TIRF studies then revealed that yeast twinfilin accelerates depolymerization at barbed ends, and together with yeast Srv2/CAP strongly accelerates depolymerization at pointed ends (Johnston et al., 2015). Single molecule imaging further showed that twinfilin directly associates with barbed ends (Fig. 4 D).

In mammals, the three twinfilin isoforms (Twf1, Twf2a, and Twf2b) all collaborate with mammalian CAP1 to accelerate barbed end depolymerization, similar to their yeast counterparts (Hilton et al., 2018). However, unlike the yeast proteins, mammalian twinfilins and CAP only modestly accelerate pointed end depolymerization. While there is some evidence to suggest that twinfilin interacts processively at barbed ends (Johnston et al., 2015), these data are far from conclusive, and there have been other observations pointing instead to a non-processive mechanism in which each twinfilin molecule removes only one to two subunits per binding event at the barbed end (Mwangangi et al., 2021). The effects of twinfilin on barbed end depolymerization also depend on the nucleotide state (or “age”) of the filament, as twinfilin accelerates the depolymerization of ADP-P_i_, but slows the depolymerization of ADP F-actin (Shekhar et al., 2021). Further, these activities are observed even under assembly-promoting conditions, i.e., in the presence of actin monomers. Thus, twinfilin appears to be a specialized depolymerase, capable of bypassing the normal filament aging step in disassembly in vitro. It is possible that these unique activities of twinfilin are employed in vivo to accelerate the disassembly of newly polymerized regions of cellular actin networks.

The ability of cofilin to accelerate filament depolymerization was validated in 2017 by two groups employing in vitro microfluidics-assisted TIRF microscopy (Shekhar and Carlier, 2017; Wioland et al., 2017). Earlier evidence from in vitro bulk assays had argued that cofilin increases the rate of subunit dissociation from pointed ends (Carlier et al., 1997). However, in the absence of direct visualization of these effects, this model remained controversial, especially since severing had been observed in EM and TIRF studies. However, the studies above ended this debate by directly observing barbed and pointed ends of filaments shortening at an accelerated rate in the presence of all three mammalian cofilin isoforms. Interestingly, once filaments became heavily decorated along their sides by cofilin, only the pointed ends continued to depolymerize faster than control filaments, whereas the barbed ends depolymerized more slowly than control filaments (Wioland et al., 2017, 2019a). Furthermore, under these conditions (saturating cofilin) the barbed ends depolymerized persistently, even in the presence of actin monomers or capping protein. Differences in the depolymerization rates were observed for cofilin isoforms. ADF accelerated depolymerization by as much as 22-fold at pointed ends (Fig. 4 D), whereas cofilin-1 and cofilin-2 had only two- to threefold effects on depolymerization at pointed ends. These differences between ADF and cofilin-1 could possibly explain why many non-muscle cell types express both isoforms (Hotulainen et al., 2005; Vartiainen et al., 2002).

In 2019, two groups used microfluidics-assisted TIRF to show that Srv2/CAP synergizes with cofilin in depolymerizing the pointed ends of filaments (Kotila et al., 2019; Shekhar et al., 2021). These studies showed that N-CAP alone accelerates pointed end depolymerization by approximately fivefold, but together with cofilin induces depolymerization >300-fold faster than control filaments, reaching rates of ∼50 subunits s^−1^. These striking effects were observed at concentrations of CAP and cofilin similar to their cellular concentrations. Further, the mechanism involved CAP molecules processively tracking the pointed ends of cofilin-decorated filaments (Fig. 4 D). The pointed end depolymerization effects induced by yeast twinfilin and CAP may involve a related mechanism. Consistent with this idea, mutations in the conserved actin-binding surface of the HFD domain of CAP abolish its in vitro ability to jointly catalyze pointed end depolymerization with either twinfilin or cofilin (Johnston et al., 2015; Shekhar et al., 2019). The crystal structure of a ternary HFD-actin-cofilin complex further suggests that the pointed end depolymerization effects of CAP may require interactions of HFD domains with both actin subunits exposed at the pointed end (Kotila et al., 2019). In vivo, the robust pointed end depolymerization mechanism of CAP and cofilin could be used to rapidly depolymerize the F-actin fragments resulting from severing and/or to maintain rapid turnover at one end of a cellular actin network decorated by tropomyosin, which protects filament sides from severing (DesMarais et al., 2002; Gateva et al., 2017; Jansen and Goode, 2019; Ostrowska et al., 2017; Robaszkiewicz et al., 2016). Indeed, mutations in the HFD that disrupt CAP depolymerization activities cause striking defects in actin organization and dynamics in vivo (Chaudhry et al., 2013; Toshima et al., 2016).

## Step 5: Actin monomer recycling

Once ADP-actin monomers are released from filament ends (catalyzed by severing and depolymerization mechanisms), they must be recycled back to the ATP-bound state to be used for new rounds of filament assembly. Released ADP-actin monomers that are free, i.e., not bound to other factors, spontaneously undergo fast nucleotide exchange (ATP for ADP). However, due to the high affinities of cofilin and twinfilin for ADP-actin monomers, a substantial fraction of released ADP-actin monomers in the cytosol is predicted to be bound to cofilin and twinfilin (Blanchoin and Pollard, 1999; Goode et al., 1998; Nishida, 1985). Importantly, cofilin and twinfilin also strongly suppress nucleotide exchange on actin monomers, as does thymosin β4, which is a highly abundant peptide in the cytosol of animal cells (Safer et al., 1991). How then, under these cytosolic conditions, are actin monomers recycled?

Two cellular factors, profilin and Srv2/CAP, have been implicated in overcoming these barriers to promote nucleotide exchange and recharging of monomers (Fig. 4 E; Balcer et al., 2003; Mattila et al., 2004; Mockrin and Korn, 1980; Nishida, 1985; Pantaloni and Carlier, 1993). Both proteins are abundant and conserved throughout the plant, animal, and fungal kingdoms (Karlsson and Dráber, 2021; Ono, 2013). Srv2/CAP is more effective than profilin in promoting nucleotide exchange on cofilin-bound ADP-actin monomers in vitro (Balcer et al., 2003; Chaudhry et al., 2010; Kotila et al., 2018). This can be explained by the low affinity of profilin for ADP-actin monomers (Nishida, 1985; Ojala et al., 2002; Perelroizen et al., 1995). In contrast, Srv2/CAP exhibits a high affinity for ADP-actin monomers (K_d_ ∼20 nM), and catalyzes cofilin dissociation from monomers (Balcer et al., 2003; Bertling et al., 2007; Chaudhry et al., 2007, 2010, 2014; Jansen et al., 2014; Mattila et al., 2004; Nomura and Ono, 2013; Quintero-Monzon et al., 2009). These activities of Srv2/CAP are mediated by its C-CAP, consisting of actin-binding WH2 and β-sheet/CAP and X-linked retinitis pigmentosa 2 protein (CARP) domains (Fig. 3; Chaudhry et al., 2010; Jansen et al., 2014; Makkonen et al., 2013; Moriyama and Yahara, 2002). A cocrystal structure of the C-CAP dimer bound to two actin monomers revealed the structural basis for CAP-accelerated nucleotide exchange on actin (Kotila et al., 2018). Interestingly, the C-terminal tail of C-CAP (last four residues) directly binds to the nucleotide-sensing region of G-actin. Deletion of these residues abolishes C-CAP effects on nucleotide exchange in vitro and leads to pronounced defects in yeast cell growth and actin organization. C-CAP also contains a profilin-binding polyproline tract (P1), which binds directly to profilin (Bertling et al., 2007). This interaction may facilitate Srv2/CAP’s “middleman” role in converting cofilin- and twinfilin-bound ADP-actin monomers into profilin-bound ATP-actin monomers ready for new rounds of assembly (Fig. 4 E; Mattila et al., 2004; Quintero-Monzon et al., 2009). Consistent with this idea, a recent study determined that CAP is the limiting factor for monomer recycling and sustained motility in a “closed” in vitro reconstitution system containing purified proteins (verproline central acidic on beads, plus actin, profilin, CP, Arp2/3 complex, cofilin, and CAP; Colin et al., 2023).

One important question for the future is how the actin monomer recycling functions of Srv2/CAP are integrated with its F-actin severing and depolymerization functions (discussed in the previous section). The filament severing and depolymerization activities of Srv2/CAP are mediated by its N-CAP, whereas monomer recycling is mediated by its C-CAP. Interestingly, the two halves of Srv2/CAP can function in trans as independent fragments in vitro and in vivo (Chaudhry et al., 2014). Thus, the F-actin and G-actin turnover functions of Srv2/CAP appear to be fairly autonomous. This raises the intriguing question of why these two functions are linked in one molecule and whether physically joining the two halves of Srv2/CAP provides intramolecular regulation between these two functions and/or new as-yet-to-be-discovered activities.

Another key question is how the monomer pool is controlled in vivo. Cells maintain a large fraction of their total actin in a monomeric state (∼100 µM [Koestler et al., 2009]) so that high concentrations of monomers are available to drive rapid polymerization. In vertebrate cells, the largest fraction of the actin monomer pool is bound to thymosin-β4, owing to its high abundance (Devineni et al., 1999; Hannappel and Leibold, 1985; Weber et al., 1992). Thymosin-β4 blocks actin monomer addition to filament ends, i.e., is a sequestering protein. Importantly, thymosin-β4 interactions with monomers are highly transient and thus actin monomers can be rapidly handed off to profilin, which is not a sequestering protein (Pantaloni and Carlier, 1993). Profilin-bound monomers can be added to free barbed ends or used by formins and Ena/vasodilator-stimulated phosphoprotein to accelerate filament elongation. Therefore, due to the rapid exchange of actin monomers between thymosin-β4 and profilin, cells are able to maintain a homeostatic balance between their large pool of thymosin-β4–sequestered monomers (80–90 µM) and smaller pool of assembly-ready profilin-bound monomers (10–20 µM; Fig. 4 E; Carlier and Shekhar, 2017; Pollard and Cooper, 1984).

## Deployment of actin turnover mechanisms in vivo

The mechanisms discussed above provide cells with a diverse toolkit for reshaping actin network architecture and tuning network turnover rates. For instance, branched networks at the leading edge of cells and sites of endocytosis start out as densely arborized arrays, but as the networks move inward, they are pruned (by debranchers like cofilin, coronin, and GMF), transforming their filamentous organization and properties. At the same time, some of these networks undergo severing and depolymerization, reducing average filament length. More work is needed to determine how network architecture is dynamically transformed in vivo. Indeed, a recent study showed that CAP, enhanced by its interactions with Abp1, dynamically slides and bundles actin filaments (branched and unbranched), coalescing them into compact bundles through a novel activity (Guo et al., 2023). This study also showed that specific mutations in CAP that disrupt these activities cause strong defects in actin organization in vivo. This is likely to be only one of many remodeling activities used in cells to dynamically reorganize actin networks.

How different turnover mechanisms are used in vivo may also depend on whether a network is earmarked for the dynamic maintenance or the demolition pathway (Fig. 2). Dynamic maintenance requires balanced assembly and disassembly at the two ends of the network, regardless of whether the turnover rate is slow or fast. However, we still do not understand how this balance is achieved. On the other hand, the demolition pathway requires strong inhibition of network assembly (Fig. 2). This may be achieved by locally shutting off actin nucleators and polymerases and by activating barbed end cappers and depolymerizers, coupled with filament severing and depolymerization. There is some evidence for this in filopodial retraction, as cofilin and twinfilin appear at filopodial tips just before they retract (Breitsprecher et al., 2011; Rzadzinska et al., 2009). Similarly, lamellipodial retraction is accompanied by the appearance of many disassembly-promoting factors, including cofilin, AIP1, coronin, Srv2/CAP, GMF, and twinfilin (Bertling et al., 2007; Cai et al., 2007; Goode et al., 2018; Hakala et al., 2021; Iwasa and Mullins, 2007; Svitkina, 2018; Vartiainen et al., 2000).

Another future challenge is to determine how the different actin-disassembly mechanisms are influenced by the presence of other actin-binding proteins decorating networks. For instance, tropomyosins (TPMs) are an abundant family of coiled-coil proteins that polymerize along the sides of filaments, decorating most known cellular actin structures (Gunning et al., 2015). Mammalian cells express >40 different TPM isoforms generated by alternative splicing of four distinct genes. These isoforms exhibit a wide range of antagonistic effects on cofilin in vitro (Gateva et al., 2017; Ostrowska et al., 2017), which is thought to provide cells with the plasticity to differentially protect various actin structures. Indeed, specific roles for TPM isoforms are suggested by their distinct in vivo localization patterns to actin networks with different turnover dynamics and lifetimes (Gunning et al., 2005). Cofilin can be antagonized in vitro by other members of the ADF-H protein family (e.g., Abp1, drebrin, and coactosin), which compete with cofilin for F-actin–side binding (Poukkula et al., 2011). This view is supported by genetic observations showing that loss of drebrin or coactosin leads to increased cofilin-mediated F-actin turnover (Grintsevich and Reisler, 2014; Kim et al., 2014). Mechanisms that accelerate pointed end depolymerization are also likely to be influenced by actin-binding proteins that cap and/or polymerize pointed ends, such as tropomodulins and VopF (Almenar-Queralt et al., 1999; Kudryashova et al., 2022; Rao et al., 2014). Thus, there is still much to learn about how these and other side-binding proteins affect the multicomponent disassembly mechanisms discussed above.

Finally, it remains to be determined how actin network remodeling and turnover are differentially affected by the crosslinking proteins that spatially organize filaments into distinct higher-order networks. For instance, fascin and fimbrin bundle filaments into tightly packed linear arrays, e.g., in filopodia. Some studies suggest that fascin bundling enhances severing by cofilin (Breitsprecher et al., 2011; Christensen et al., 2017), while other studies suggest that fascin blocks cofilin severing (Chikireddy et al., 2023, *Preprint*). Other longer-lived cellular actin structures are organized by a different set of crosslinkers (e.g., α-actinin, filamin, espin, calponin, and Eps8), which may endow networks with different degrees of resistance or vulnerability to specific disassembly mechanisms. Ultimately, understanding how various cellular actin networks (e.g., lamellipodia, filopodia, microvilli, stereocilia, and actin cables) are differentially remodeled and turned over will require a deeper knowledge of how their filament side-binding and crosslinking proteins influence the disassembly mechanisms, combined with how the activities of disassembly factors are locally regulated in the vicinity of each network.

## Concluding remarks

Over the past two decades, our understanding of how cellular actin networks are disassembled has expanded profoundly due to a large body of work defining the structures, in vitro mechanisms, and biological roles of the disassembly and turnover machinery. One of the most important insights made is that distinct sets of disassembly factors work together in multicomponent mechanisms to produce emergent effects, as discussed above. These advances notwithstanding, more work is needed to define the structural basis of each mechanism and to determine which mechanisms are used to drive the turnover of which actin networks in vivo. Further, we only have a limited understanding of how these turnover mechanisms are regulated by signaling pathways through posttranslational modification (PTM) of actin and actin-binding proteins. Ultimately, gaining a more complete understanding of cellular actin network disassembly will require developing and combining diverse approaches, including high-resolution cell imaging, structural biology, in vitro multiwavelength single molecule imaging, optogenetics, theory, and modeling.
